# Correlation of interleukin-6 with Epstein–Barr virus levels in COVID-19

**DOI:** 10.1186/s13054-020-03384-6

**Published:** 2020-11-23

**Authors:** Georg Franz Lehner, Sebastian Johann Klein, Heinz Zoller, Andreas Peer, Romuald Bellmann, Michael Joannidis

**Affiliations:** 1 Division of Intensive Care and Emergency Medicine, Department of Internal Medicine, Medical University Innsbruck, Anichstraße 35, 6020 Innsbruck, Austria; 2 Internal Medicine I, Department of Internal Medicine, Medical University Innsbruck, Anichstraße 35, 6020 Innsbruck, Austria

**Keywords:** COVID-19, EBV, IL-6, Inflammation, Coronavirus disease 2019, SARS-CoV-2, Severe acute respiratory syndrome coronavirus 2, Epstein–Barr virus, Interleukin-6

## Introduction

Severe acute respiratory syndrome coronavirus 2 (SARS-CoV-2) causes coronavirus disease 2019 (COVID-19) pneumonia with respiratory failure in a subset of infected patients. To date, it is unclear which factors trigger or cause the severe course of disease. Moreover, there is only limited evidence concerning extrapulmonary manifestations of COVID-19.

We observed that COVID-19 patients invasively ventilated in our intensive care unit (ICU) showed biochemical abnormalities that resemble hepatitis and pancreatitis typically caused by herpesviruses like Epstein–Barr virus (EBV) or cytomegalia virus (CMV). Moreover, a subgroup of COVID-19 patients exhibit a hyperinflammatory pattern similar to secondary hemophagocytic lymphohistiocytosis (sHLH) [1, 2], a syndrome that can be triggered by viruses like EBV.

Thus, we speculated whether critically ill COVID-19 patients show evidence of EBV- or CMV-infection or reactivation and quantified EBV as well as CMV DNA levels in blood by PCR.

## Case series

Herein, we report a retrospective analysis using data of the Tyrolean COVID-19 intensive care registry. We evaluated all COVID-19 patients that were treated between March 26, 2020, and April 20, 2020, in the Medical ICU at the Medical University Innsbruck, Austria, due to respiratory failure and required invasive ventilation (*n* = 20). Eighteen patients had at least one EBV and CMV PCR during ICU stay and were thus eligible for analysis. They were compared to eighteen consecutive invasively ventilated ICU patients without COVID-19.

We found that 78% of COVID-19 patients had EBV viremia, 39% even above 1000 IU/ml. Prevalence and levels of EBV viremia were significantly higher in COVID-19 patients compared to non-COVID-19 patients (44.4%, Pearson Chi-square *p* = 0.040, Mann–Whitney *U* test *p* = 0.022, SPSS 26 (IBM, Armonk, NY)).

In contrast, only 17% of COVID-19 patients and 5.6% of non-COVID-19 patients had evidence of CMV viremia, which was not significantly different between the groups (Pearson Chi-square *p* = 0.289). No correlations between viral load of EBV and blood levels of hepatic and pancreatic enzymes or cholestasis parameters were detected.

**Fig. 1:**
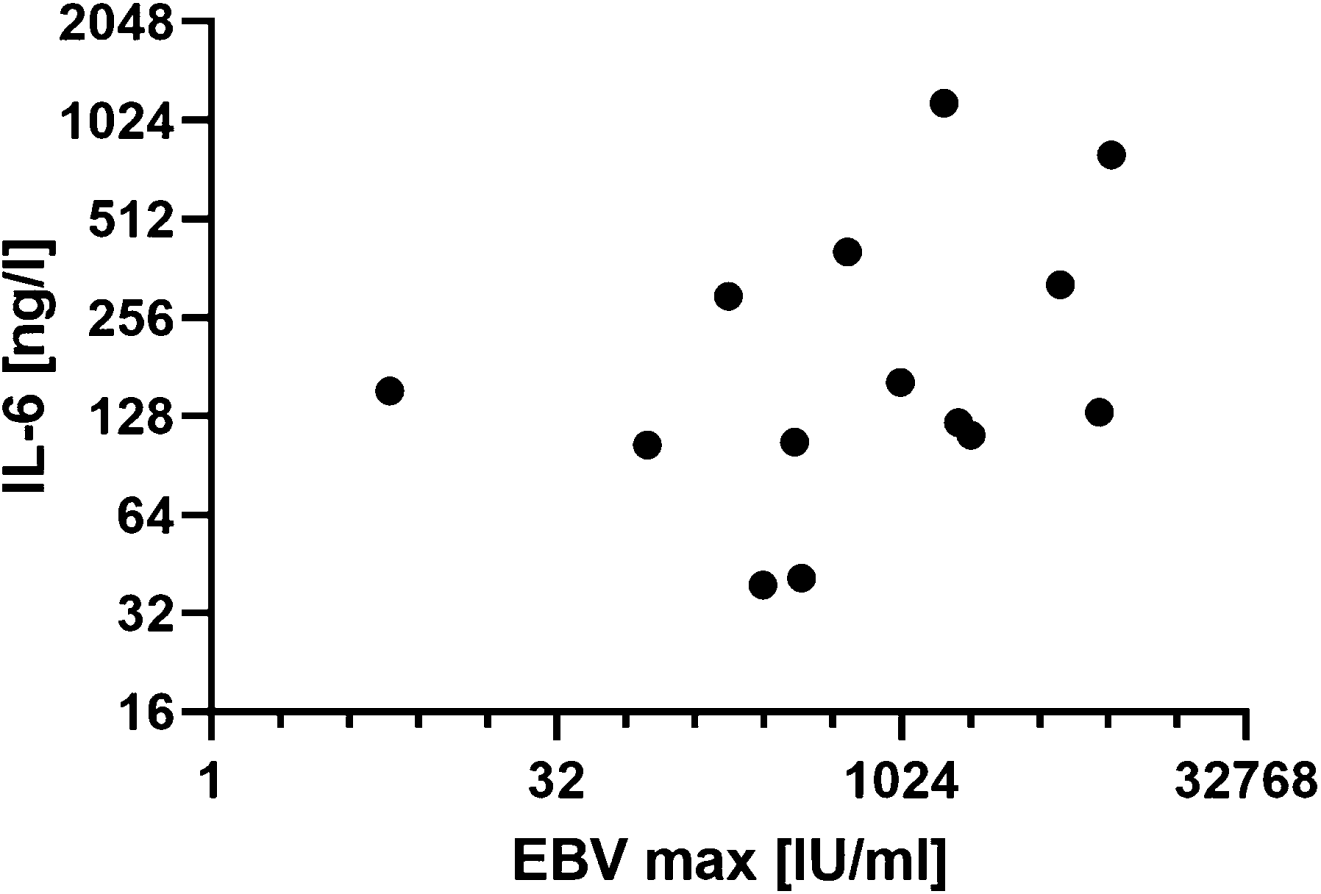
Corresponding blood levels of Epstein–Barr virus (EBV) DNA and interleukin-6 (IL-6) in critically ill coronavirus disease 2019 (COVID-19) patients

However, there was a significant correlation between EBV viremia and interleukin-6 (IL-6) level (Fig. 1, *r* = 0.621, *p* = 0.006) in COVID-19 patients, but not in non-COVID-19 patients (*r* = − 0.195, *p* = 0.438, Spearman’s rank-order correlation). Detailed patient characteristics are outlined in Table 1.

**Table 1:** Overview of parameters (median (25th–75th percentiles) and # median (25th percentile)) between coronavirus disease 2019 (COVID-19) patients with and without EBV viremia and non-COVID-19 patients

	COVID-19 (*n* = 18)	COVID-19 EBV negative (*n* = 4)	COVID-19 EBV positive (*n* = 14)	Non-COVID-19 (*n* = 18)
Age (years)	60.5 (52.0–64.5)	45.5 (43.3–59.0)	61.5 (53.8–66.8)	58.8 (47.8–72.3)
IL-6 (ng/l)	125.1 (40.5–302.8)	20.9 (18.0–101.4)	142.0 (106.0–342.4)	85.7 (43.6–377.4)
CRP (mg/dl)	14.6 (4.3–16.6)	5.8 (2.2–14.8)	15.5 (7.7–19.7)	8.3 (3.7–28.1)
PCT (µg/l)	0.3 (0.2–0.8)	0.2 (0.1–0.4)	0.4 (0.2–1.1)	1.3 (0.2–6.8)
Bilirubin total (mg/dl)	0.9 (0.4–1.2)	0.67 (0.4–0.9)	1.0 (0.4–1.3)	0.7 (0.5–2.6)
ASAT (U/l)	67.5 (33.5–91.8)	63.0 (18.8–114.0)	67.5 (33.5–85.8)	51.0 (38.5–122.3)
ALAT (U/l)	49.0 (37.5–80.5)	73.5 (20.5–185.75)	49.0 (40.5–72.3)	56.0 (23.8–194.8)
GGTP (U/l)	173.0 (61.5–370.0)	206.5 (8.8–657.0)	151.5 (60.3–370.0)	131.5 (76.5–305.0)
AP (U/l)	103.5 (79.0–222.0)	131.0 (102.3–273.8)	93.0 (57.3–222.0)	220.5 (175.5–559.3)
Amylase (U/l)	37.0 (24.5–67.0)	37.0 (22.0)#	43.0 (23.3–71.8)	39.0 (15.0–48.0)
Lipase (U/l)	43.0 (21.0–75.0)	26.0 (26.0)#	47.5 (19.3–74.0)	30.0 (16.0–66.0)

EBV, Epstein–Barr virus; ICU, intensive care unit; IL-6, interleukin-6; PCT, procalcitonin; CRP, c-reactive protein; ASAT, aspartate aminotransferase; ALAT, alanine aminotransferase; GGTP, gamma glutamyltransferase; AP, alkaline phosphatase

## Discussion

This is the first systematic report of EBV viremia in critically ill COVID-19 patients which revealed two important findings: First, COVID-19 patients have a higher prevalence of EBV viremia compared to non-COVID-19 patients. Second, levels of EBV viremia correlate with IL-6 in COVID-19 patients but not in non-COVID-19 patients.

Since EBV can induce immune dysregulation and expression of IL-6 in peripheral blood mononuclear cells (PBMCs) via deoxyuridine triphosphate nucleotidohydrolase (dUTPase) in vitro [3], one might speculate that EBV acts as an additional inflammatory trigger in critically ill COVID-19 patients.

The observation that two patients without history of allergy but an EBV viremia above 1000 IU/ml developed a generalized maculopapular rash following administration of amoxicillin/clavulanate and piperacillin/tazobactam, further emphasizes the hypothesized immunological impact of EBV in this setting [4, 5].

Although this observation was made in a limited number of patients in a retrospective analysis, the systematic approach based on registry data minimizes the risk of selection bias. Moreover, we compared COVID-19 patients to an appropriate control group. The findings concerning EBV and CMV viremia in the control group are in accordance with previously reported cumulative incidences (i.e., 48% and 18%, respectively) [6].

## Conclusion

These data suggest that EBV viremia is highly prevalent in COVID-19 patients with respiratory failure and associated with systemic inflammation as evidenced by high IL-6 levels. It remains to be elucidated whether EBV viremia represents an epiphenomenon in COVID-19 or plays a pathogenetic role as additional trigger of a systemic inflammatory response in this setting.

## Data Availability

No data are publicly available at this time.

## Abbreviations

SARS-CoV-2: Severe acute respiratory syndrome coronavirus 2
COVID-19: Coronavirus disease 2019
EBV: Epstein–Barr virus
CMV: Cytomegalia virus
sHLH: Secondary hemophagocytic lymphohistiocytosis
ICU: Intensive care unit
IL-6: Interleukin-6
PBMCs: Peripheral blood mononuclear cells
dUTPase: Deoxyuridine triphosphate nucleotidohydrolase
PCT: Procalcitonin
CRP: C-reactive protein
ASAT: Aspartate aminotransferase
ALAT: Alanine aminotransferase
GGTP: Gamma glutamyltransferase
AP: Alkaline phosphatase
